# The state of vestibular neurology: Diagnostic challenges and emerging opportunities

**DOI:** 10.1177/00368504261484700

**Published:** 2026-09-03

**Authors:** Diego Kaski, Richard Ibitoye

**Affiliations:** 1 SENSE Research Unit, Department of Clinical and Movement Neurosciences, 4919 UCL Institute of Neurology, London, UK; 2 1982 Department of Neurology, North Bristol NHS Trust, Bristol, UK

**Keywords:** vestibular neurology, dizziness, vertigo, acute vestibular syndrome, vestibular migraine, bilateral vestibulopathy, functional dizziness, small vessel disease

## Abstract

Vestibular neurology has undergone substantial evolution over the past two decades, driven by advances in bedside diagnostics, neuroimaging, computational neuroscience, and therapeutics. Despite this progress, dizziness and vertigo remain among the most common yet diagnostically challenging neurological complaints. This narrative review examines the current state of vestibular neurology, focusing on key clinical syndromes, ongoing diagnostic controversies, and emerging opportunities for innovation. Particular attention is given to acute vestibular syndrome, audiovestibular disorders, bilateral vestibulopathy, functional dizziness, vestibular migraine, and the role of cerebral small vessel disease. In parallel, advances in understanding vestibular contributions to cognition and the development of novel therapeutic strategies, including vestibular implants, neuromodulation, and digital diagnostics, are highlighted. The field is at a pivotal stage, where integration of technology, improved phenotyping, and mechanistic insights promise to transform both diagnosis and management.

## Introduction

Vestibular neurology occupies a unique position at the intersection of neurology, otology, audiology, and rehabilitation medicine. It is concerned with patients presenting with dizziness, vertigo, and imbalance, symptoms that are common, heterogeneous, and often diagnostically non-specific. These symptoms may arise from peripheral vestibular disorders, central nervous system pathology, systemic disease, or functional mechanisms, posing a substantial challenge to clinicians.

Dizziness is broadly defined as a disturbed or impaired sense of spatial orientation without a false or distorted sense of motion.^1^ Vertigo, in contrast, refers to the false sensation of self-motion or motion of the environment, typically (but not exclusively) rotational. Unsteadiness denotes a feeling of imbalance or instability, particularly during standing or walking. While these distinctions are conceptually useful, patients frequently struggle to articulate their symptoms, limiting their diagnostic utility in isolation.

Historically, vestibular neurology has been constrained by reliance on symptom description and relatively crude diagnostic tools. However, the past two decades have seen a paradigm shift. The development of structured bedside assessments, such as the HINTS (Head-Impulse, Nystagmus, Test-of-Skew)^2^ examination and novel diagnostic and treatment manoeuvres for positional vertigo, alongside advances in video-oculography, imaging, and computational modelling, has significantly enhanced diagnostic accuracy. At the same time, emerging insights into vestibular contributions to cognition and network-level brain function have broadened the conceptual scope of the field.

This review explores the current landscape of vestibular neurology, focusing on major clinical syndromes, persistent diagnostic challenges, and emerging opportunities for innovation. This review is guided by the Scale for the Assessment of narrative review articles.^3^ Because this is a narrative review, studies were selected to provide a clinically relevant overview rather than a systematic synthesis. Priority was given to consensus statements, landmark studies, high-quality systematic reviews, and recent advances published over approximately the past decade, together with seminal earlier work where necessary to provide historical context. Literature was identified through searches of PubMed and reference lists using combinations of terms related to vestibular neurology, dizziness, vertigo, vestibular migraine, functional dizziness, bilateral vestibulopathy, vestibular cognition, and acute vestibular syndrome. Rather than providing an exhaustive catalogue of vestibular disorders, this review focuses on areas that illustrate major shifts in contemporary vestibular neurology, including advances in bedside diagnosis, evolving disease mechanisms, and emerging therapeutic opportunities. Together these topics highlight how vestibular medicine is moving from symptom-based classification towards mechanism-informed neurological practice.

Unlike previous reviews, which have largely focused on individual vestibular disorders or specific diagnostic techniques, this review aims to provide an integrated neurological perspective on the field. By bringing together advances in acute diagnosis, immune-mediated disease, functional disorders, vestibular migraine, cerebral network disorders, spatial cognition, and emerging neurotechnologies, we highlight common themes that are reshaping vestibular neurology. In particular, we emphasise the transition from symptom-based diagnosis towards mechanism-based classification, the growing importance of systems neuroscience, and opportunities for precision diagnostics and targeted therapies.

## Acute vestibular syndrome

Acute vestibular syndrome (AVS), characterized by the rapid onset of persistent (>24 hours) vertigo, nausea, vomiting, gait instability, and spontaneous nystagmus, represents one of the most critical diagnostic scenarios in vestibular neurology. The primary challenge lies in distinguishing usually benign peripheral causes, such as acute unilateral vestibulopathy, from potentially life-threatening central causes, most notably posterior circulation stroke. Approximately one quarter of patients presenting with AVS ultimately have a central cause, usually ischemic stroke,^4^ yet diagnostic errors remain common because focal neurological signs may be absent in up to two-thirds of cases.^5^

Traditional approaches to the assessment of dizziness relied heavily on symptom quality, attempting to classify patients according to whether they described “vertigo”, “dizziness”, “light-headedness”, or “imbalance”. However, evidence suggests that symptom descriptors are often inconsistent and have limited diagnostic utility.^6^ Patients frequently struggle to accurately characterize their symptoms, and clinicians may interpret terminology differently. Greater emphasis has therefore shifted toward a structured history focused on symptom timing and provoking factors, as embodied in approaches such as TiTrATE (Timing, Triggers and Targeted Examination).^7^ Distinguishing continuous spontaneous symptoms from episodic or positionally triggered symptoms narrows the diagnostic possibilities and guides the selection of appropriate bedside examinations.

The introduction of the HINTS examination has transformed bedside assessment, demonstrating superior sensitivity to early MRI in expert hands for identifying stroke in patients with AVS.^2^ However, its use requires careful patient selection and expertise. HINTS has been validated primarily in patients with continuous symptoms and spontaneous nystagmus, and its indiscriminate use outside this setting may reduce diagnostic accuracy.^8^ Importantly, incorporation of hearing assessment into HINTS+ increases sensitivity for detecting strokes involving the anterior inferior cerebellar artery (AICA) territory,^8^ where labyrinthine involvement may produce clinical features resembling peripheral vestibular disorders. Acute hearing loss or tinnitus in a patient with AVS should therefore not be assumed to indicate a purely inner ear process but may instead represent a vascular event involving the labyrinth or its central connections.

Overreliance on imaging also remains a major source of diagnostic error. One is the misinterpretation of a normal head CT report as excluding acute stroke.^9^ Another is not recognising that - although MRI with diffusion-weighted imaging is widely considered the reference standard for acute stroke detection – small posterior circulation strokes can be missed, particularly within the first 48 hours following symptom onset.^10,11^ Consequently, a normal early MRI should not supersede concerning clinical findings, as half of small infarcts are due to dissection or stenosis, meaning a high risk of recurrence if not treated. Rather than seeking only obvious neurological deficits, clinicians should actively evaluate for subtle oculomotor abnormalities, hearing asymmetry, and severe gait or truncal instability, which may be more informative indicators of central pathology. Severe truncal ataxia, particularly when disproportionate to other examination findings, should raise suspicion for cerebellar involvement even in the absence of spontaneous nystagmus.^8^

Acute vestibular migraine presents an additional challenge because it may closely resemble both stroke and peripheral vestibular disorders during a first presentation. Patients may report vertigo, nausea, headache, gait imbalance, photophobia, phonophobia, or motion sensitivity, often in the context of normal imaging findings. Likewise, central positional nystagmus, commonly associated with vestibular migraine, may mimic benign paroxysmal positional vertigo and contribute to diagnostic confusion.^12,13^ Recognition of these mimics requires careful history taking that emphasizes symptom timing, triggers, associated migrainous features, and targeted bedside examination.

Despite dissemination of structured diagnostic algorithms, substantial variability persists in acute vertigo assessment, reflecting differences in clinician experience and confidence with vestibular examination techniques. Emerging technologies, including video-oculography, portable eye-tracking systems, digital bedside vestibular assessment tools, and telemedicine-based pathways, may help improve diagnostic consistency by providing objective measures of eye movement abnormalities. Portable VOG may help reduce variability in the interpretation of acute eye movement findings but requires user training and has not been validated in emergency settings. Indeed, evidence increasingly suggests that targeted training, even of relatively short duration, substantially improves clinician performance in the assessment of acutely dizzy patients, emphasizing that systematic bedside evaluation remains central to improving diagnostic accuracy and reducing avoidable harms in patients presenting with AVS.

Taken together, these developments represent a broader shift away from reliance on neuroimaging alone towards integrating structured bedside examination with targeted imaging. This change has important implications not only for diagnostic accuracy but also for resource utilisation and clinician training.

## Autoimmune inner ear disease

An autoimmune basis for sensorineural hearing loss was first described in patients with progressive hearing loss who responded to immunosuppression with corticosteroids.^14^ The broader label of autoimmune inner ear disease (AIED) recognises the common involvement of vestibular sense organs and nerves.^15–17^ AIED has no reliable diagnostic tests, and diagnostic criteria have not been defined.^17^ There is an overlap with Ménière’s disease for which the mechanism of disease remains elusive,^18,19^ and may be immunological.^20^

AIED is characterised by progressive, often bilateral and asymmetric sensorineural hearing loss that evolves over weeks to months and demonstrates responsiveness to immunosuppressive therapy.^14–17^ However, the absence of specific and reliable biomarkers limits diagnostic certainty and means that AIED remains largely a diagnosis of exclusion.^17^ While most proposed diagnostic criteria focus predominantly on hearing loss,^17,21,22^ vestibular symptoms are increasingly recognised as an important and underappreciated component of disease. Vestibular dysfunction has been reported in approximately 50–60% of patients with AIED^17,23,24^ and may present heterogeneously with episodic vertigo, chronic disequilibrium, oscillopsia, gait ataxia, motion intolerance, or positional symptoms. Importantly, vestibular impairment may occasionally dominate the clinical presentation, and, in some patients, may precede significant auditory deterioration.^25^

Ménière’s disease presents with fluctuating audiovestibular symptoms, classically episodic vertigo, hearing loss, tinnitus, and aural fullness.^26^ Endolymphatic hydrops – enlargement of the endolymphatic space, which occurs with many pathologies of the inner ear,^27^ is common in Ménière’s disease.^28^ With modern neuroimaging techniques, delayed contrast-enhanced MRI can reveal imaging markers of hydrops which correlate with hearing function in Menieres.^29^ The same imaging markers have been seen in autoimmune inner ear disease as described in our own series of patients with autoimmune vestibulopathy who had both cochlear and vestibular hydrops,^25^ consistent with a shared pathological basis.

The pathophysiology of AIED itself remains incompletely understood. Proposed mechanisms include direct antibody-mediated injury to cochlear and vestibular structures, immune complex deposition within the inner ear vasculature, and T-cell mediated inflammatory responses. Multiple candidate antigens have been identified, including heat shock protein-70 (HSP70),^30^ cochlin^31^ and other inner ear proteins^32^; however, none have shown sufficient sensitivity or specificity for routine clinical use.^33^ Consequently, no universally accepted diagnostic biomarker for AIED currently exists.

Recent imaging advances have enabled in vivo visualization of endolymphatic hydrops and inflammatory inner ear changes.^34,35^ Delayed contrast-enhanced MRI techniques have demonstrated cochlear enhancement, vestibular enhancement and hydrops in selected patients with suspected autoimmune pathology.^36^ However, imaging findings must be interpreted cautiously because radiological abnormalities do not always correlate with clinical symptoms or disease severity.^37^

The management of AIED is empirical. Corticosteroids are given as first-line treatment because early intervention may prevent irreversible hearing loss, although relapse during steroid tapering is common.^38,39^ In keeping with the management of other autoimmune diseases, steroid-sparing immunosuppressive agents such as methotrexate, azathioprine, and biologic therapies have also been used.^17^ Resulting hearing loss can be treated with hearing aids, with a role for cochlear implantation for the most severe cases. With respect to vestibular hypofunction, vestibular rehabilitation is recommended to facilitate central compensation, thereby improving balance and visual stability. Future progress will likely depend on improved phenotyping of auditory and vestibular deficits in individual cases, studying of the mechanism linking immune-mediated inner ear disease to the development of hydrops, and identifying disease-specific biomarkers. The absence of validated biomarkers illustrates a broader challenge in vestibular neurology, namely that advances in understanding disease mechanisms have outpaced the development of clinically applicable diagnostic tests.

## Functional dizziness

Functional dizziness (FD) has emerged as one of the most common causes of chronic vestibular symptoms. Persistent postural-perceptual dizziness (PPPD) – the major form of functional dizziness - accounts for around 1 in 5 diagnoses in neuro-otology clinics.^40–42^ Critically, a diagnosis of PPPD and related functional disorders such as mal de debarquement syndrome (MdDS) should be made from specific symptoms such as visual motion sensitivity, meeting established diagnostic criteria.^43,44^ Dizziness occurring without evidence of structural pathology, and not meeting diagnostic criteria for any vestibular disorder, should be described as “cryptogenic”, rather than functional.

Research on functional neurological disorders has sought to ground the emergence of functional percepts or movements in the neuroscience of a hierarchical, predictive (Bayesian) model of brain function.^45^ In a hierarchical Bayesian brain, neural modules account for predictions (beliefs) and prediction errors. Sensory feedback is compared to predictions (beliefs) at lower levels of the hierarchy. Resulting prediction errors propagate to higher levels of the hierarchy. At each level, errors are used to update and ideally optimise predictions – with this updating process itself corresponding to perception. If – for any reason – a belief (prediction) becomes overvalued (unduly precise), prediction errors are maintained and ineffective updating processes contribute to a distorted or abnormal percept^46^ or action.^47^

MdDS is a transient or persistent disorder characterised by persistent non-spinning oscillatory vertigo, classically described as sensations of rocking, bobbing or swaying, following exposure to passive motion such as sea travel, flights or prolonged vehicle journeys.^44^ Unlike PPPD, in which symptoms are typically exacerbated by movement and complex visual environments, MdDS symptoms improve transiently with re-exposure to passive motion, such as driving in a car, representing a characteristic and diagnostically useful feature.^48^ The relationship between MdDS and PPPD remains incompletely understood, but both are perceptual disorders arising from multisensory integration. Similar to PPPD, routine vestibular examinations – such as eye movement testing - are commonly normal.^49–51^

Management of MdDS remains challenging and evidence for treatment is comparatively limited. Conventional vestibular rehabilitation approaches developed for peripheral vestibular disorders frequently demonstrate inconsistent benefit, perhaps reflecting the fact that the primary dysfunction appears to reside within higher-order processing of motion and spatial information rather than within the vestibular periphery itself. Current treatment approaches include symptomatic use of benzodiazepines and serotonergic agents in selected patients, together with vestibular rehabilitation strategies adapted specifically for motion perception disorders.^52^ Emerging therapies have increasingly focused on modulating central mechanisms of self-motion processing, including optokinetic stimulation paradigms designed to recalibrate maladaptive internal representations of movement and gravity.^53^ One argument is that treatment should aim not simply to suppress symptoms but to modify the brain’s internal model of space and motion. Analogous to other functional dizziness disorders such as PPPD, rehabilitation may therefore require a shift away from strategies that reinforce excessive bodily monitoring and toward approaches that encourage exposure, external attentional focus, and restoration of confidence in movement and balance.

PPPD is a functional vestibular disorder characterised by persistent non-spinning dizziness, subjective imbalance, and spatial disorientation present on most days for at least three months.^43^ PPPD is frequently precipitated by conditions that impair or threaten balance, including vestibular neuritis, benign paroxysmal positional vertigo, vestibular migraine, panic attacks, mild traumatic brain injury, and other acute medical illnesses. Symptoms are typically exacerbated by upright posture, self-motion, and visually complex or moving environments such as supermarkets, busy streets, or crowds. The term PPPD unifies features of several previously described syndromes including chronic subjective dizziness, phobic postural vertigo, visual vertigo and space-motion discomfort.

Persistence of symptoms in PPPD associates with central, rather than peripheral vestibular factors. In a prospective study of chronic functional dizziness as an outcome after vestibular neuritis, psychological and behavioural factors including autonomic arousal, increased body vigilance, visual dependence, dependent personality traits and anxiety following vestibular neuritis were more predictive of PPPD development than objective measures of vestibular dysfunction itself.^54^ Pre-existing personality and behavioural traits may therefore predispose to a maladaptive response to vestibular neuritis, from which PPPD emerges. More recent work suggests negative illness perceptions, anxiety, cognitive appraisals and perceived injustice may contribute significantly to symptom burden and disability,^55,56^ reinforcing a holistic – biopsychosocial – view of PPPD, rather than as a purely psychiatric or structural neurological disorder.

Symptoms in PPPD are proposed to arise from mechanisms shared with other functional neurological disorders, as a disturbance of predictive coding.^57^ A triggering anxiogenic event such as unilateral loss of vestibular function generates a large prediction error – unexpected head motion. A hypervigilant postural strategy – e.g. stiffening of the body – is then adopted,^58^ linked with a belief that unwanted destabilising motion has occurred. A potential advantage of a hypervigilant strategy is that this reduces the risk of being destabilised by external motion stimuli. In susceptible individuals (e.g. those with premorbid anxiety, or visual dependence)^54,59^ the related beliefs of a high risk of instability may become overvalued. In the Bayesian model, this implies prediction modules for a high-risk postural strategy become unduly precise. As a result, otherwise innocuous sensory stimuli – such as those arising from spontaneous sway due to sensorimotor noise – continue to be interpreted as potential evidence of unexpected motion, producing a persistent percept of unsteadiness/dizziness.^60^ Although predictive coding has become the dominant theoretical framework for understanding PPPD, it should be regarded as a mechanistic model rather than a proven explanation. Future work will need to determine how computational accounts relate to neurophysiological biomarkers and treatment response.

Behavioural work suggests a deficit in prediction is core to PPPD. In a paradigm where participants were asked to reproduce postural sway experienced on a challenging standing balance task, reproduced sway in patients with PPPD was larger than expected, being double the observed sway.^61^ Importantly, observed sway was also less than in controls, as a result of the hypervigilant, stiffened, postural strategy. This mismatch was not seen in healthy controls or patients with bilateral vestibular hypofunction. This finding of postural misperception suggests symptoms in PPPD arise not from excessive bodily movements, but from inaccurate percepts of the size and salience of those movements. Within the predictive coding framework, otherwise innocuous postural sensory signals are thus processed as significant prediction errors. The very behavioural adaptations which reduce body sway in high-risk scenarios and thus minimise prediction error may – in the longer term – impede recovery. This is because a stiff posture with minimal sway prevents larger postural sensory signals which are needed to update maladaptive postural predictions towards more adaptive predictions. The combination of both reduced sway and greater reported instability in PPPD is noteworthy and should be explored as a potential behavioural biomarker for the disorder.

An important clinical challenge lies in differentiating PPPD from disorders with overlapping symptom profiles, particularly vestibular migraine.^62^ More than half of patients with PPPD fulfil criteria for migraine headaches,^63^ and substantial overlap exists between visual sensitivity, motion intolerance and dizziness symptoms. Rather than being mutually exclusive disorders, PPPD and vestibular migraine share overlapping mechanisms and frequently coexist.

Management of PPPD requires a positive diagnosis and clear communication regarding underlying mechanisms. Explaining that symptoms arise from altered perceptual processing rather than structural damage can improve diagnostic confidence and engagement with treatment. Indeed, explanation is often itself therapeutic.^64^ Current treatment strategies focus on disrupting maladaptive balance behaviours through vestibular rehabilitation, cognitive behavioural approaches, and serotonergic medications where indicated.^65^ Emerging approaches, including virtual reality-based therapies and interventions targeting altered self-motion perception may further improve outcomes.

## Vestibular migraine

Vestibular migraine (VM) is now recognised as the most common cause of spontaneous episodic vertigo and one of the most frequent diagnoses encountered in dizziness clinics. Population studies suggest a prevalence approaching 1–3%, although the condition remains substantially underdiagnosed owing to its heterogeneous clinical presentation and lack of objective biomarkers. VM occupies an important position at the interface between headache medicine and vestibular neurology because symptoms often extend beyond classical migraine manifestations and may mimic both peripheral and central vestibular disorders.

The clinical syndrome is characterised by recurrent episodes of vestibular symptoms of moderate or severe intensity lasting between 5 minutes and 72 hours.^66^ Vertigo may be spontaneous or positional and can include rotational vertigo, head-motion induced dizziness, rocking sensations, imbalance, or spatial disorientation. Episodes are frequently accompanied by migrainous features including headache, photophobia, phonophobia, visual aura, nausea, and motion sensitivity, although headache itself may be absent during attacks. Between attacks, patients frequently report persistent symptoms including visual dependence, sensitivity to complex visual environments, motion intolerance and subjective disequilibrium. Neurological examination is typically normal in the interictal period; however, during acute episodes spontaneous or positional nystagmus may be present and may occasionally resemble benign paroxysmal positional vertigo (BPPV), contributing to diagnostic uncertainty. Central positional nystagmus is increasingly recognised as an important clinical manifestation of VM and may reflect disturbances of central vestibular processing rather than peripheral labyrinthine dysfunction.^13^

Current diagnostic criteria, jointly developed by the Bárány Society and International Headache Society, remain entirely clinically based.^66^ Definite VM requires: (i) at least five episodes of vestibular symptoms of moderate or severe intensity lasting between 5 minutes and 72 hours; (ii) a current or previous history of migraine; (iii) migrainous features accompanying at least 50% of vestibular episodes; and (iv) symptoms not better accounted for by another vestibular or headache disorder. A category of probable vestibular migraine is recognised for patients fulfilling only some criteria. Although these diagnostic criteria demonstrate reasonable long-term reliability, they nevertheless remain limited by the absence of a biological gold standard and by substantial overlap with conditions including Ménière’s disease, PPPD, and recurrent positional vertigo syndromes.

The pathophysiology of VM remains incompletely understood, although increasing evidence supports the concept of a disorder of central multisensory integration involving a distributed vestibulo-thalamo-cortical network rather than a primary peripheral vestibular disorder. Traditional migraine mechanisms involving trigeminovascular activation and neuropeptide release, particularly calcitonin gene-related peptide (CGRP), probably contribute to symptom generation. However, these mechanisms alone do not adequately explain the vestibular and spatial symptoms characteristic of VM. Instead, emerging evidence suggests widespread abnormalities in processing and integrating vestibular, visual, somatosensory, autonomic and nociceptive information. Structural and functional imaging studies have implicated regions including the thalamus, insular cortex, temporo-parietal cortex and vestibular association areas as key nodes within this network.^67^

Experimental studies additionally suggest altered visuo-vestibular interactions as a fundamental feature of VM. Patients demonstrate heightened visual dependence and abnormal responses to visual motion stimuli,^68^ findings that align closely with the commonly reported symptoms of visually induced dizziness, spatial disorientation and self-motion sensitivity. Abnormal vestibular perceptual thresholds and exaggerated effects of visual motion exposure^69^ suggest that cortical mechanisms responsible for integrating visual and vestibular signals may become dysregulated, leading to altered estimates of self-motion and orientation in space. Such abnormalities may explain why patients frequently describe sensations of rocking, tilting or disequilibrium even when conventional vestibular testing is normal.

These observations have led to the emerging conceptualisation of VM as a network disorder of central integration. Rather than reflecting dysfunction of a single anatomical structure, symptoms likely arise from abnormal interactions between distributed cortical and subcortical systems responsible for motion perception, spatial orientation and sensory prediction. This framework also helps explain the broad clinical spectrum of VM, including visual motion sensitivity, motion sickness susceptibility, positional symptoms and its frequent coexistence with functional vestibular disorders such as PPPD. The absence of reliable biomarkers probably reflects the episodic and distributed nature of the disorder, with abnormalities emerging from altered interactions across large-scale brain networks rather than structural lesions within a single anatomical site. Developing biomarkers capable of capturing these transient network changes therefore remains an important research priority.

Treatment of VM remains challenging because the evidence base is considerably weaker than for migraine headache and many recommendations are extrapolated from migraine management more generally.^70^ Treatment must always start with education, an explanation of the underlying cause of a patient’s symptoms, in language that can be easily understood by the patient.

Acute treatment commonly includes antiemetics, vestibular suppressants, non-steroidal anti-inflammatory drugs, and triptans, although evidence supporting the efficacy of triptans specifically for vestibular symptoms remains limited and uncertain. Preventive pharmacological therapies include beta-blockers, calcium channel blockers, antidepressants, antiepileptic drugs and CGRP-targeted therapies. To date, eight studies have assessed a range of CGRP-targeted therapies and gepants and generally reported improvement of vestibular symptoms and scores used but have included small sample sizes and lack blinding and control groups. Data on anti-CGRP treatments for VM remain limited, and efficacy established in headache migraine cannot be straightforwardly extrapolated to VM given differences in underlying pathophysiology. Recent systematic reviews indicate that evidence supporting pharmacological interventions remains low or very low in certainty and no single agent can currently be recommended preferentially.^70^ Consequently, treatment selection often remains guided by co-existing symptoms and patient factors; for example, venlafaxine may be preferred where anxiety or PPPD coexist, while topiramate may be useful in patients with obesity or frequent headache burden.

Non-pharmacological measures remain important and include regular sleep patterns, hydration, exercise, dietary modification, trigger identification, and management of psychological comorbidities. Vestibular rehabilitation may benefit selected patients, particularly those with persistent visual dependence or interictal imbalance, although current evidence remains limited.^70^ As understanding of the central network mechanisms underlying VM improves, future treatments may increasingly target sensory integration and cortical processing rather than migraine pathways alone.

## Cerebral small vessel disease related dizziness

Cerebral small vessel disease (cSVD) is increasingly recognised as a contributor to chronic dizziness and imbalance in older adults. cSVD represents the commonest acquired cerebral disorder in later life and encompasses a spectrum of pathological processes affecting small perforating arteries, arterioles and capillaries, leading to white matter hyperintensities, lacunes, cerebral microbleeds and disruption of white matter integrity.^71^ Although cSVD is classically associated with cognitive decline, gait disturbance and falls, increasing evidence suggests that dizziness and impaired spatial orientation may represent early clinical manifestations of network dysfunction resulting from diffuse white matter injury.

Dizziness in older adults frequently remains unexplained despite detailed vestibular and neurological investigation. Up to half of older adults presenting with dizziness have normal peripheral vestibular testing and no specific structural diagnosis can be identified.^72^ Historically such syndromes have been described by various terms including presbyvertigo, presbyataxia, disequilibrium of ageing, or unexplained dizziness in the elderly. More recently, evidence has suggested that such symptoms may arise not from peripheral vestibular failure but from disruption of distributed neural systems involved in balance control and spatial orientation.

Current conceptual models propose that cSVD-related dizziness reflects a disorder of network connectivity,^73^ and the term MicroAngiopathic Induced Dizziness (MAID) has been proposed. White matter injury may impair communication between cortical and subcortical regions involved in vestibular processing, gait control and multisensory integration. Several mechanisms have been proposed. Disconnection of frontal gait networks and basal ganglia circuits may impair the planning and execution of postural responses; disruption of vestibular cortical networks involving temporo-parietal and insular regions may alter spatial orientation and self-motion perception; and impaired integration between intended movement and sensory re-afference may produce a mismatch between expected and actual movement signals, generating subjective sensations of imbalance or dizziness.

Neuroimaging studies increasingly support this model. Older adults with “unexplained dizziness” demonstrate greater white matter disease burden and reduced integrity of frontal white matter pathways compared with asymptomatic controls.^74^ Particularly important associations have been observed in frontal deep white matter, the genu of the corpus callosum, and widespread bihemispheric white matter networks previously implicated in gait and executive function. Importantly, vestibular reflex and perceptual measures often remain normal, suggesting that symptoms arise from disturbances in higher-order processing rather than impairment of peripheral vestibular function itself.^74^

Neurophysiological studies further suggest that cSVD alters cortical mechanisms involved in postural control. Electroencephalographic recordings obtained during standing demonstrate abnormal cortical responses in older dizzy patients, including altered oscillatory activity and changes in network connectivity associated with postural instability. Greater white matter disease burden appears to be associated with increased neural resource demands during balance tasks,^75^ suggesting that cortical systems increasingly compensate for declining processing efficiency. Furthermore, exploratory studies combining functional near-infrared spectroscopy with balance perturbation paradigms have demonstrated greater prefrontal activation in individuals with greater cSVD burden despite relatively preserved behavioural measures of balance performance, supporting the idea that compensatory cortical recruitment occurs before overt clinical impairment becomes apparent.

Importantly, these observations suggest that dizziness may represent an early manifestation of cerebral network dysfunction before conventional neurological deficits become apparent. If confirmed prospectively, dizziness could emerge as a clinically useful marker of early cerebral small vessel disease.

Recent work additionally suggests that subjective dizziness may reflect altered awareness of normally unconscious postural processes. During standing, healthy individuals continuously generate small corrective responses to maintain balance. Older adults with chronic dizziness appear to demonstrate altered sensorimotor cortical activity during these subtle balance perturbations, potentially reflecting increased top-down monitoring of postural signals and heightened sensitivity to instability.^76^ Such findings may help explain why many patients describe persistent unsteadiness despite minimal objective abnormalities on clinical examination.

Together, these findings suggest that microangiopathic-induced dizziness may be understood as a disorder of distributed network dysfunction rather than focal vestibular pathology.^77^ This framework also helps reconcile the common clinical observation that symptoms of imbalance and spatial disorientation often precede objective abnormalities on neurological examination. Future work combining advanced neuroimaging, neurophysiological measures and behavioural assessments may help identify biomarkers of early disease and provide opportunities for targeted interventions before the emergence of more overt gait and cognitive decline. These findings expand the concept of vestibular neurology beyond the labyrinth to distributed cerebral networks.

## The vestibular system and spatial cognition

The vestibular system contributes not only to reflexive control of eye movements and posture, but also to higher-order cognitive functions involved in constructing an internal representation of the body in space. Spatial cognition encompasses the neural processes responsible for perceiving, encoding and updating information regarding self-location, body orientation, navigation and spatial awareness. Rather than operating as an isolated sensory system, vestibular signals are integrated with visual, proprioceptive and motor information to generate coherent representations of the external world and the position of the body within it.

A defining characteristic of vestibular cortical processing is that vestibular signals project widely to multimodal areas, for example in the parietal, temporo-parietal, insular and frontal cortices.^78–80^ These networks support self-motion perception, spatial orientation and body awareness through continuous integration of multiple sensory inputs. Consequently, vestibular dysfunction often manifests as disturbances of orientation and perception rather than purely balance impairment.^81^ Recognition that vestibular dysfunction influences cognition as well as balance represents one of the most important conceptual developments in modern vestibular neurology.

## Future directions and emerging opportunities

**Figure 1.**
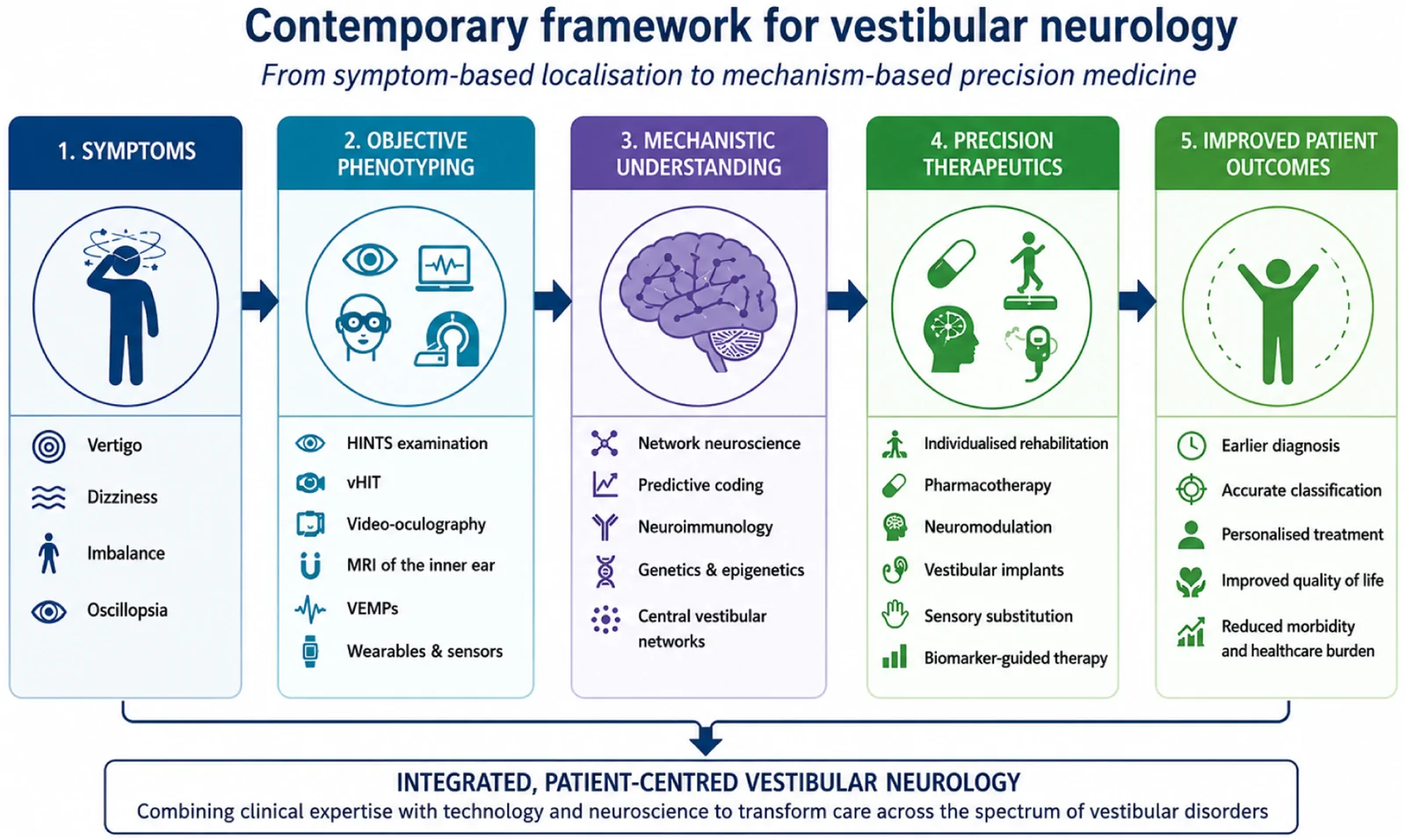
Contemporary framework for vestibular neurology. Vestibular neurology has evolved from a traditional approach based on symptoms and localisation to a modern framework that integrates objective phenotyping with advances in systems neuroscience, immunology, genetics, and neurotechnology. This enables mechanism-based diagnosis and precision therapeutics, ultimately improving patient outcomers. We acknowledge the use of ChatGPT (OpenAI, GPT-4) to assist in the design of this figure.

Several common themes emerge from the preceding sections. Across disorders, advances in vestibular neurology increasingly reflect improvements in disease phenotyping, objective measurement, and mechanistic understanding rather than discovery of entirely new syndromes (Figure 1). The next decade of vestibular medicine is likely to be defined by advances in precision diagnostics and the development of targeted therapies. Historically, management of vestibular disorders has focused predominantly on symptom reduction and rehabilitation. However, recent developments in genetics, immunology and neurotechnology suggest a transition toward biologically informed disease classification and mechanism-based treatment approaches.

Collectively, the preceding sections illustrate that vestibular neurology is no longer defined simply by disorders of the labyrinth or brainstem. Instead, the field increasingly encompasses distributed brain networks, immune-mediated disease, computational models of perception, genetics, and neurotechnology. Viewing these developments together reveals common mechanistic themes that may not be apparent when individual disorders are considered in isolation.

Objective vestibular phenotyping is likely to become increasingly important. Advances in wearable sensors, portable video-oculography, quantitative gait analysis, machine learning-based eye movement interpretation, and multimodal imaging offer opportunities to improve diagnostic consistency and identify disease-specific biomarkers. However, most technologies currently demonstrate high diagnostic performance only within narrowly defined clinical contexts (Table 1), and future work should prioritise external validation across diverse patient populations before widespread clinical implementation.

**Table 1.** Contemporary diagnostic technologies in vestibular neurology.

Technology	Best validated application	Strength	Important limitation
HINTS	Acute vestibular syndrome	Greater sensitivity than early MRI in expert hands	Requires expertise
vHIT	Peripheral vestibular hypofunction	High specificity for semicircular canal dysfunction	Normal in vestibular migraine and many central disorders
VEMPs	Otolith disorders	Detect utricular/saccular dysfunction	Limited disease specificity
Delayed gadolinium MRI	Ménière’s disease/hydrops	Visualises endolymphatic hydrops	Hydrops not disease-specific
Video-oculography	Acute eye movement disorders	Objective quantification	Interpretation expertise needed
AI eye-tracking (emerging)	Stroke screening	Promising	Limited prospective validation

### Vestibular ataxia and emerging genetic discoveries

One of the most important developments has been increasing recognition of disorders combining vestibular and cerebellar dysfunction. Previously considered rare, vestibular ataxias are increasingly recognised owing to advances in neurogenetics and improvements in vestibular phenotyping. These disorders frequently present with gait imbalance, oscillopsia and combined ocular motor abnormalities that may be overlooked if vestibular and cerebellar systems are considered separately.

The discovery of biallelic repeat expansions in *RFC1* as a cause of cerebellar ataxia, neuropathy and vestibular areflexia syndrome (CANVAS) has substantially altered the diagnostic landscape of late-onset ataxia.^82^ *RFC1* expansions appear to represent one of the commonest identifiable genetic causes of previously unexplained adult-onset ataxia and have highlighted the importance of incorporating vestibular testing into the assessment of cerebellar syndromes. Similarly, identification of *FGF14* repeat expansions causing SCA27B has established another relatively common cause of late-onset cerebellar disease with prominent vestibular and oculomotor manifestations.^83^ Recognition of these disorders has immediate clinical implications because some symptoms, including downbeat nystagmus and gait impairment, may be responsive to pharmacological intervention with aminopyridines.

Future work will likely focus on refining genotype–phenotype relationships and identifying disease-modifying approaches. Greater use of long-read sequencing technologies and broader implementation of genomic screening in vestibular clinics may reveal additional repeat expansion disorders and improve diagnostic yield in unexplained dizziness syndromes.

### Immunological mechanisms and precision medicine

A second major area of development concerns the role of immune mechanisms in vestibular disease. Menière’s disease has increasingly been viewed as a heterogeneous disorder rather than a single disease entity, with evidence suggesting multiple clinical and biological subtypes, including immune-associated forms. Associations with migraine, allergy and immune-mediated conditions support a multifactorial pathophysiology involving both environmental and genetic influences.

Advances in genomic methods have identified several candidate genes associated with familial Menière’s disease, including *OTOG*, *TECTA* and *MYO7A*,^84^ suggesting complex inheritance patterns and raising the possibility of biologically distinct endophenotypes.

Parallel progress is occurring in autoimmune inner ear disease (AIED), where increasing recognition of inflammatory pathways raises the possibility of biomarker-driven treatment approaches. Identification of immune signatures may eventually enable stratification of patients likely to benefit from corticosteroids, biologic therapies or other targeted immunomodulatory treatments.^20^ Future studies combining genomics, proteomics and immune profiling may permit a transition from symptom-based classifications toward molecularly defined vestibular disorders.

### Neuroprosthetics and sensory substitution

Technological innovation is also beginning to reshape treatment approaches for patients with severe vestibular loss. Bilateral vestibular loss remains associated with substantial disability despite conventional vestibular rehabilitation, with persistent imbalance, oscillopsia and falls contributing significantly to impaired quality of life.

One promising approach is sensory substitution through wearable vibrotactile devices. The BalanceBelt provides information regarding trunk orientation using tactile feedback delivered around the waist, thereby substituting for missing vestibular signals.^85^ Studies in severe bilateral vestibular loss have demonstrated improvements in self-reported mobility and balance, with sustained adherence over periods exceeding two years.^86^

Although these findings are encouraging, most studies to date have originated from groups involved in device development and evaluation. Independent validation using objective outcome measures remains important. To address this, our group is currently planning an independent prospective study of vibrotactile sensory substitution in bilateral vestibular loss, assessing quantitative mobility, balance and patient-centred outcomes separately from the device manufacturer.

Perhaps the most transformative development is the vestibular implant.^87^ Analogous to cochlear implants, vestibular implants use motion sensors and surgically implanted electrodes to stimulate vestibular pathways directly. Early studies have demonstrated improvements in posture, gait and oscillopsia in selected patients with severe bilateral vestibular loss.^88^ However, the technology remains investigational, and important challenges remain regarding surgical optimisation, preservation of hearing, signal coding strategies and long-term outcomes.

### Limitations

As a narrative review, this article has several limitations. It does not employ a systematic search strategy or formal assessment of study quality and therefore cannot provide quantitative estimates of effect or certainty of evidence. Topic selection inevitably reflects the authors’ judgement regarding areas of greatest contemporary relevance, and some emerging fields may receive greater emphasis than more established areas. Nevertheless, the narrative approach allows integration of evidence across traditionally separate disciplines - including neurology, neuro-otology, cognitive neuroscience, immunology, and rehabilitation - to provide a conceptual overview of a rapidly evolving field.

## Conclusion

As vestibular neurology continues to evolve, an important challenge extends beyond scientific and technological advances, and concerns education itself. Despite dizziness and balance disorders representing some of the most common neurological symptoms encountered in clinical practice, structured training in vestibular neurology remains limited in many parts of the world. Diagnostic uncertainty, under-recognition of vestibular syndromes, and reduced confidence with bedside examination persist across specialities, contributing to delays in diagnosis and avoidable morbidity. Expanding access to vestibular education and fostering greater integration of vestibular medicine within neurology training programmes will therefore be essential to meet the growing clinical burden of these disorders.

Ultimately, vestibular neurology occupies a distinctive position within medicine. Dizziness, vertigo and disorders of balance affect not only movement and spatial orientation but can profoundly disrupt an individual’s confidence, independence, identity and interaction with the world around them. Symptoms are often invisible, difficult to articulate and frequently misunderstood, leaving many patients feeling isolated or dismissed. In this context, perhaps more than in many other areas of clinical neuroscience, there is an opportunity for the human side of medicine to emerge. Careful listening, taking time to explain diagnoses and mechanisms, and recognising the social, emotional, and psychological factors that shape illness experience can have a transformative effect. As our biological understanding and therapeutic options continue to advance, maintaining this human dimension will remain equally important, because in vestibular medicine some of the greatest gains may arise not only from new technologies and treatments, but also from helping patients feel understood.

## References

[1] Bisdorff A Von Brevern M Lempert T , et al. Classification of vestibular symptoms: towards an international classification of vestibular disorders. J Vestib Res 2009; 19: 1–13. 10.3233/VES-2009-0343 19893191

[2] Kattah JC Talkad AV Wang DZ , et al. HINTS to diagnose stroke in the acute vestibular syndrome: three-step bedside oculomotor examination more sensitive than early MRI diffusion-weighted imaging. Stroke 2009; 40: 3504–3510. 10.1161/STROKEAHA.109.551234 19762709 PMC4593511

[3] Baethge C Goldbeck-Wood S Mertens S . SANRA-a scale for the quality assessment of narrative review articles. Res Integr Peer Rev 2019; 4: 5. 10.1186/s41073-019-0064-8 30962953 PMC6434870

[4] Hotson JR Baloh RW . Acute vestibular syndrome. N Engl J Med 1998; 339: 680–685. 10.1056/NEJM199809033391007 9725927

[5] Saber Tehrani AS Kattah JC Mantokoudis G , et al. Small strokes causing severe vertigo: frequency of false-negative MRIs and nonlacunar mechanisms. Neurology 2014; 83: 169–173. 10.1212/WNL.0000000000000573 24920847 PMC4117176

[6] Newman-Toker DE Cannon LM Stofferahn ME , et al. Imprecision in patient reports of dizziness symptom quality: a cross-sectional study conducted in an acute care setting. Mayo Clin Proc 2007; 82: 1329–1340. 10.4065/82.11.1329 17976352

[7] Newman-Toker DE Edlow JA . TiTrATE: A Novel, Evidence-Based Approach to Diagnosing Acute Dizziness and Vertigo. Neurol Clin 2015; 33: 577–599. 10.1016/j.ncl.2015.04.011 26231273 PMC4522574

[8] Anburajan G Kattah J Wang Z , et al. HINTS+ to diagnose AICA stroke: systematic review of the diagnostic impact of acute unilateral hearing loss in the acute vestibular syndrome. J Neurol 2026; 273: 131. 10.1007/s00415-026-13641-3 41665728 PMC13668945

[9] Savitz SI Caplan LR Edlow JA . Pitfalls in the diagnosis of cerebellar infarction. Acad Emerg Med 2007; 14: 63–68. 10.1197/j.aem.2006.06.060 17200515

[10] Oppenheim C Stanescu R Dormont D , et al. False-negative diffusion-weighted MR findings in acute ischemic stroke. AJNR Am J Neuroradiol 2000; 21: 1434–1440. 11003275 PMC7974060

[11] Choi J-H Oh EH Park M-G , et al. Early MRI-negative posterior circulation stroke presenting as acute dizziness. J Neurol 2018; 265: 2993–3000. 10.1007/s00415-018-9097-z 30341546

[12] Bertholon P Bronstein AM Davies RA , et al. Positional down beating nystagmus in 50 patients: cerebellar disorders and possible anterior semicircular canalithiasis. J Neurol Neurosurg Psychiatry 2002; 72: 366–372. 10.1136/jnnp.72.3.366 11861698 PMC1737794

[13] Lemos J Strupp M . Central positional nystagmus: an update. J Neurol 2022; 269: 1851–1860. 10.1007/s00415-021-10852-8 34669008

[14] McCabe BF . Autoimmune sensorineural hearing loss. Ann Otol Rhinol Laryngol 1979; 88: 585–589. 10.1177/000348947908800501 496191

[15] Ruckenstein MJ . Autoimmune inner ear disease. Curr Opin Otolaryngol Head Neck Surg 2004; 12: 426–430. 10.1097/01.moo.0000136101.95662.aa 15377956

[16] Bovo R Ciorba A Martini A . The diagnosis of autoimmune inner ear disease: evidence and critical pitfalls. Eur Arch Otorhinolaryngol 2009; 266: 37–40. 10.1007/s00405-008-0801-y 18777037

[17] Ciorba A Corazzi V Bianchini C , et al. Autoimmune inner ear disease (AIED): A diagnostic challenge. Int J Immunopathol Pharmacol 2018; 32: 2058738418808680. 10.1177/2058738418808680 30376736 PMC6213300

[18] Rizk HG Mehta NK Qureshi U , et al. Pathogenesis and etiology of Ménière disease: A scoping review of a century of evidence: A scoping review of a century of evidence. JAMA Otolaryngol Head Neck Surg 2022; 148: 360–368. 35142800 10.1001/jamaoto.2021.4282

[19] Mohseni-Dargah M Falahati Z Pastras C , et al. Meniere’s disease: Pathogenesis, treatments, and emerging approaches for an idiopathic bioenvironmental disorder. Environ Res 2023; 238: 116972. 10.1016/j.envres.2023.116972 37648189

[20] Yuan VG Xia A Santa Maria PL . Immunological mechanisms in Meniere’s disease. Front Immunol 2025; 16: 1639916. 10.3389/fimmu.2025.1639916 40936890 PMC12420328

[21] Campbell KC Klemens JJ . Sudden hearing loss and autoimmune inner ear disease. J Am Acad Audiol 2000; 11: 361–367. 10976497

[22] García-Berrocal JR Trinidad A Ramírez-Camacho R , et al. Immunologic work-up study for inner ear disorders: looking for a rational strategy. Acta Otolaryngol 2005; 125: 814–818. 10.1080/00016480510038059 16158526

[23] Agrup C Luxon LM . Immune-mediated inner-ear disorders in neuro-otology. Curr Opin Neurol 2006; 19: 26–32. 10.1097/01.wco.0000194143.02171.46 16415674

[24] Brant JA Eliades SJ Ruckenstein MJ . Systematic review of treatments for autoimmune inner ear disease. Otol Neurotol 2015; 36: 1585–1592. 10.1097/MAO.0000000000000875 26485595

[25] Mendis S Longley N Morley S , et al. Autoimmune vestibulopathy-A case series. Brain Sci 2022; 12: 306. 10.3390/brainsci12030306 35326263 PMC8946225

[26] Sajjadi H Paparella MM . Meniere’s disease. Lancet 2008. https://www.thelancet.com/journals/lancet/article/PIIS0140673608611617/abstract 10.1016/S0140-6736(08)61161-7 18675691

[27] Schuknecht HF Gulya AJ . Endolymphatic hydrops. An overview and classification. Ann Otol Rhinol Laryngol 1983; 106: 1–20. 10.1177/00034894830920s501 6414357

[28] Schuknecht HF Suzuka Y Zimmermann C . Delayed endolymphatic hydrops and its relationship to Meniére’s disease. Ann Otol Rhinol Laryngol 1990; 99: 843–853. 10.1177/000348949009901101 2241008

[29] Del Poggio A Cangiano I Baldissera E , et al. Clinical significance of endolymphatic hydrops on MRI in Cogan’s syndrome: a case series of five patients. Eur Arch Otorhinolaryngol 2025; 282: 2753–2760. 10.1007/s00405-024-09191-x 39891695

[30] Shin SO Billings PB Keithley EM , et al. Comparison of anti-heat shock protein 70 (anti-hsp70) and anti-68-kDa inner ear protein in the sera of patients with Meniere’s disease. Laryngoscope 1997; 107: 222–227. 10.1097/00005537-199702000-00015 9023247

[31] Baruah P . Cochlin in autoimmune inner ear disease: is the search for an inner ear autoantigen over? Auris Nasus Larynx 2014; 41: 499–501. 10.1016/j.anl.2014.08.014 25199741

[32] Mulry E Parham K . Inner ear proteins as potential biomarkers. Otol Neurotol 2020; 41: 145–152. 10.1097/MAO.0000000000002466 31789810

[33] Matsuoka AJ Harris JP . Autoimmune inner ear disease: a retrospective review of forty-seven patients. Audiol Neurootol 2013; 18: 228–239. 10.1159/000351289 23817208

[34] Nakashima T Naganawa S Sugiura M , et al. Visualization of endolymphatic hydrops in patients with Meniere’s disease. Laryngoscope 2007; 117: 415–420. 10.1097/MLG.0b013e31802c300c 17279053

[35] Nakashima T Naganawa S Teranishi M , et al. Endolymphatic hydrops revealed by intravenous gadolinium injection in patients with Ménière’s disease. Acta Otolaryngol 2010; 130: 338–343. 10.1080/00016480903143986 19685358

[36] Lobo D Tuñón M Villarreal I , et al. Intratympanic gadolinium magnetic resonance imaging supports the role of endolymphatic hydrops in the pathogenesis of immune-mediated inner-ear disease. J Laryngol Otol 2018; 132: 554–559. 10.1017/S0022215118000749 29888688

[37] Xie W Shu T Liu J , et al. The relationship between clinical characteristics and magnetic resonance imaging results of Ménière disease: a prospective study. Sci Rep 2021; 11: 7212. 10.1038/s41598-021-86589-1 33785791 PMC8010013

[38] Alexander TH Weisman MH Derebery JM , et al. Safety of high-dose corticosteroids for the treatment of autoimmune inner ear disease. Otol Neurotol 2009; 30: 443–448. 10.1097/MAO.0b013e3181a52773 19395984

[39] Pathak S Hatam LJ Bonagura V , et al. Innate immune recognition of molds and homology to the inner ear protein, cochlin, in patients with autoimmune inner ear disease. J Clin Immunol 2013; 33: 1204–1215. 10.1007/s10875-013-9926-x 23912888 PMC3809107

[40] Adamec I Juren Meaški S Krbot Skorić M , et al. Persistent postural-perceptual dizziness: Clinical and neurophysiological study. J Clin Neurosci 2020; 72: 26–30. 10.1016/j.jocn.2020.01.043 31948878

[41] Kim H-J Lee J-O Choi J-Y , et al. Etiologic distribution of dizziness and vertigo in a referral-based dizziness clinic in South Korea. J Neurol 2020; 267: 2252–2259. 10.1007/s00415-020-09831-2 32300888

[42] Xue H Chong Y Jiang ZD , et al. Etiological analysis on patients with vertigo or dizziness. Zhonghua Yi Xue Za Zhi 2018; 98: 1227–1230. 10.3760/cma.j.issn.0376-2491.2018.16.008 29747309

[43] Staab JP Eckhardt-Henn A Horii A , et al. Diagnostic criteria for persistent postural-perceptual dizziness (PPPD): Consensus document of the committee for the Classification of Vestibular Disorders of the Bárány Society. J Vestib Res 2017; 27: 191–208. 10.3233/VES-170622 29036855 PMC9249299

[44] Cha Y-H Baloh RW Cho C , et al. Mal de débarquement syndrome diagnostic criteria: Consensus document of the Classification Committee of the Bárány Society. J Vestib Res 2020; 30: 285–293. 10.3233/VES-200714 32986636 PMC9249277

[45] Edwards MJ Adams RA Brown H , et al. A Bayesian account of ‘hysteria. Brain 2012; 135: 3495–3512. 10.1093/brain/aws129 22641838 PMC3501967

[46] Pareés I Brown H Nuruki A , et al. Loss of sensory attenuation in patients with functional (psychogenic) movement disorders. Brain 2014; 137: 2916–2921. 10.1093/brain/awu237 25161293

[47] Lin D Castro P Edwards A , et al. Dissociated motor learning and de-adaptation in patients with functional gait disorders. Brain 2020; 143: 2594–2606. 10.1093/brain/awaa190 32779724

[48] Van Ombergen A Van Rompaey V Maes LK , et al. Mal de debarquement syndrome: a systematic review. J Neurol 2016; 263: 843–854. 10.1007/s00415-015-7962-6 26559820 PMC4859840

[49] Brown JJ Baloh RW . Persistent mal de debarquement syndrome: a motion-induced subjective disorder of balance. Am J Otolaryngol 1987; 8: 219–222. 10.1016/s0196-0709(87)80007-8 3631419

[50] Gordon CR Spitzer O Doweck I , et al. Clinical features of Mal de debarquement: Adaptation and habituation to sea Conditions1. J Vestib Res 1995; 5: 363–369. 8528477

[51] Cha Y-H Brodsky J Ishiyama G , et al. Clinical features and associated syndromes of mal de debarquement. J Neurol 2008; 255: 1038–1044. 10.1007/s00415-008-0837-3 18500497 PMC2820362

[52] Kinkhabwala CM Yuen E Brennan E , et al. Treatment options in Mal de débarquement syndrome: A Scoping Review. Otol Neurotol 2023; 44: e197–e203. 10.1097/mao.0000000000003832 36791362

[53] Dai M Cohen B Smouha E , et al. Readaptation of the vestibulo-ocular reflex relieves the mal de debarquement syndrome. Front Neurol 2014; 5: 124. 10.3389/fneur.2014.00124 25076935 PMC4097942

[54] Cousins S Kaski D Cutfield N , et al. Predictors of clinical recovery from vestibular neuritis: a prospective study. Ann Clin Transl Neurol 2017; 4: 340–346. 10.1002/acn3.386 28491901 PMC5420806

[55] Gamble R Sumner P Wilson-Smith K , et al. Using interpretative phenomenological analysis to probe the lived experiences of persistent postural-perceptual dizziness (PPPD). J Vestib Res 2023; 33: 89–103. 10.3233/ves-220059 36710692 PMC10041438

[56] Sereda A Lam JC Hazar A-M , et al. Psychological variables mediate symptoms in persistent Postural-Perceptual Dizziness (PPPD): A cross-sectional self-report study. Eur J Neurol 2025; 32: e70339. 10.1111/ene.70339 40853180 PMC12376309

[57] Lyndon S . Precision dynamics of predictive coding in functional neurological disorder. Brain 2026: awag101. 10.1093/brain/awag101 41823406

[58] Staab JP . Persistent postural-perceptual dizziness: Review and update on key mechanisms of the most common functional neuro-otologic disorder. Neurol Clin 2023; 41: 647–664. 37775196 10.1016/j.ncl.2023.04.003

[59] Arshad Q Cousins S Golding JF , et al. Factors influencing clinical outcome in vestibular neuritis - A focussed review and reanalysis of prospective data. J Neurol Sci 2023; 446: 120579. 10.1016/j.jns.2023.120579 36807973

[60] Popkirov S Staab JP Stone J . Persistent postural-perceptual dizziness (PPPD): a common, characteristic and treatable cause of chronic dizziness. Pract Neurol 2018; 18: 5–13. 10.1136/practneurol-2017-001809 29208729

[61] San PME Bancroft MJ Koohi N , et al. Postural misperception: a biomarker for persistent postural perceptual dizziness. J Neurol Neurosurg Psychiatry 2023; 94: 165–166. 10.1136/jnnp-2022-329321 35995549

[62] Moreno-Ajona D . Persistent postural-perceptual dizziness versus vestibular migraine: A narrative review. Headache 2026; 66: 298–306. 10.1111/head.15078 41147266 PMC12849528

[63] Sarna B Risbud A Lee A , et al. Migraine features in patients with persistent postural-perceptual dizziness. Ann Otol Rhinol Laryngol 2021; 130: 1326–1331. 10.1177/00034894211007233 33813915 PMC8487433

[64] Pollak L Klein C Stryjer R , et al. Phobic postural vertigo: a new proposed entity. IMAJ-RAMAT GAN- 2003; 5: 720–723. 14719468

[65] Trinidade A Cabreira V Kaski D , et al. Treatment of persistent postural-perceptual dizziness (PPPD). Curr Treat Options Neurol 2023; 25: 281–306. 10.1007/s11940-023-00761-8

[66] Lempert T Olesen J Furman J , et al. Vestibular migraine: diagnostic criteria. J Vestib Res 2012; 22: 167–172. 10.3233/VES-2012-0453 23142830

[67] Huang T-C Wang S-J Kheradmand A . Vestibular migraine: An update on current understanding and future directions. Cephalalgia 2020; 40: 107–121. 10.1177/0333102419869317 31394919

[68] Agarwal K Bronstein AM Faldon ME , et al. Visual dependence and BPPV. J Neurol 2012; 259: 1117–1124. 10.1007/s00415-011-6311-7 22113702

[69] Bednarczuk NF Bonsu A Ortega MC , et al. Abnormal visuo-vestibular interactions in vestibular migraine: a cross sectional study. Brain 2019; 142: 606–616. 10.1093/brain/awy355 30759189 PMC6391603

[70] Smyth D Britton Z Murdin L , et al. Vestibular migraine treatment: a comprehensive practical review. Brain 2022; 145: 3741–3754. 10.1093/brain/awac264 35859353 PMC9679161

[71] Wardlaw JM Smith C Dichgans M . Small vessel disease: mechanisms and clinical implications. Lancet Neurol 2019; 18: 684–696. 10.1016/S1474-4422(19)30079-1 31097385

[72] Ahmad H Cerchiai N Mancuso M , et al. Are white matter abnormalities associated with ‘unexplained dizziness. J Neurol Sci 2015; 358: 428–431. 10.1016/j.jns.2015.09.006 26412160 PMC4640145

[73] Kaski D Rust HM Ibitoye R , et al. Chapter 16 - Theoretical framework for “unexplained” dizziness in the elderly: The role of small vessel disease. In: Ramat S Shaikh AG (eds). Progress in Brain Research. Elsevier, 2019, pp. 225–240. 10.1016/bs.pbr.2019.04.009 31239134

[74] Ibitoye RT Castro P Cooke J , et al. A link between frontal white matter integrity and dizziness in cerebral small vessel disease. NeuroImage Clin 2022; 35: 103098. 10.1016/j.nicl.2022.103098 35772195 PMC9253455

[75] Ibitoye RT Castro P Desowska A , et al. Small vessel disease disrupts EEG postural brain networks in ‘unexplained dizziness in the elderly. Clin Neurophysiol 2021. 10.1016/j.clinph.2021.07.027 PMC8559782 34583117

[76] EllmersTJ IbitoyeR CastroP , et al. Chronic dizziness in older adults: Disrupted sensorimotor EEG beta oscillations during postural instability. Clinical Neurophysiology 2025; 174: 31–36. 10.1016/j.clinph.2025.03.03240198974

[77] Castro P Ibitoye R Ellmers T , et al. Towards an explanation for ‘unexplained’ dizziness in older people. Age Ageing 2024; 53: afae137. 10.1093/ageing/afae137 38965033 PMC11223895

[78] Lopez C Blanke O . The thalamocortical vestibular system in animals and humans. Brain Res Rev 2011; 67: 119–146. 10.1016/j.brainresrev.2010.12.002 21223979

[79] Cullen KE . Vestibular processing during natural self-motion: implications for perception and action. Nat Rev Neurosci 2019; 20: 346–363. 10.1038/s41583-019-0153-1 30914780 PMC6611162

[80] Ibitoye RT Mallas E-J Bourke NJ , et al. The human vestibular cortex: functional anatomy of OP2, its connectivity and the effect of vestibular disease. Cereb Cortex 2022. 10.1093/cercor/bhac085 PMC9890474 35235642

[81] Arshad Q Nigmatullina Y Siddiqui S , et al. Influence of biases in numerical magnitude allocation on human prosocial decision making. J Neurophysiol 2017; 118: 3007–3013. 10.1152/jn.00372.2017 28904100 PMC5712664

[82] Cortese A Simone R Sullivan R , et al. Biallelic expansion of an intronic repeat in RFC1 is a common cause of late-onset ataxia. Nat Genet 2019; 51: 649–658. 10.1038/s41588-019-0372-4 30926972 PMC6709527

[83] Rafehi H Read J Szmulewicz DJ , et al. An intronic GAA repeat expansion in FGF14 causes the autosomal-dominant adult-onset ataxia SCA50/ATX-FGF14. Am J Hum Genet 2023; 110: 105–119. 10.1016/j.ajhg.2022.11.015 36493768 PMC9892775

[84] Parra-Perez AM Lopez-Escamez JA . Types of inheritance and genes associated with familial Meniere disease. J Assoc Res Otolaryngol 2023; 24: 269–279. 10.1007/s10162-023-00896-0 37022572 PMC10335989

[85] Kingma H Felipe L Gerards M-C , et al. Vibrotactile feedback improves balance and mobility in patients with severe bilateral vestibular loss. J Neurol 2019; 266: 19–26. 10.1007/s00415-018-9133-z 30519776 PMC6722250

[86] Kingma H Hougaard DD van de Berg R . Subconscious vibrotactile stimulation improves mobility and balance in patients with bilateral vestibulopathy: adherence over 2 years. Front Neurol 2024; 15: 1491195. 10.3389/fneur.2024.1491195 39440249 PMC11494678

[87] van de Berg R Ramos A van Rompaey V , et al. The vestibular implant: Opinion statement on implantation criteria for research. J Vestib Res 2020; 30: 213–223. 10.3233/VES-200701 32651339 PMC9249290

[88] Chow MR Ayiotis AI Schoo DP , et al. Posture, gait, quality of life, and hearing with a vestibular implant. N Engl J Med 2021; 384: 521–532. 10.1056/nejmoa2020457 33567192 PMC8477665

